# Duodenal mucosal resurfacing with a GLP-1 receptor agonist increases postprandial unconjugated bile acids in patients with insulin-dependent type 2 diabetes

**DOI:** 10.1152/ajpendo.00337.2021

**Published:** 2021-12-27

**Authors:** Suzanne Meiring, Emma C. E. Meessen, Annieke C. G. van Baar, Frits Holleman, Max Nieuwdorp, Steven W. Olde Damink, Frank G. Schaap, Fred M. Vaz, Albert K. Groen, Maarten R. Soeters, Jacques J. G. H. M. Bergman

**Affiliations:** ^1^Department of Gastroenterology and Hepatology, Amsterdam University Medical Center, Amsterdam, The Netherlands; ^2^Department of Endocrinology and Metabolism, Amsterdam University Medical Center, Amsterdam, The Netherlands; ^3^Department of Internal Medicine, Amsterdam University Medical Center, Amsterdam, The Netherlands; ^4^Department of Cardiovascular Medicine, Amsterdam University Medical Center, Amsterdam, The Netherlands; ^5^Department of Surgery, NUTRIM School of Nutrition and Translational Research in Metabolism, Maastricht University, Maastricht, The Netherlands; ^6^Department of General, Visceral and Transplantation Surgery, RWTH University Hospital Aachen, Aachen, Germany; ^7^Laboratory Genetic Metabolic Diseases, Department of Clinical Chemistry and Pediatrics, Amsterdam Gastroenterology Endocrinology Metabolism, Amsterdam UMC, University of Amsterdam, Amsterdam, The Netherlands; ^8^Department of Core Facility Metabolomics, Amsterdam UMC, Amsterdam, The Netherlands

**Keywords:** bile acids, diabetes type 2, DMR, duodenal ablation, duodenal mucosal resurfacing

## Abstract

Duodenal mucosal resurfacing (DMR) is a new endoscopic ablation technique aimed at improving glycemia and metabolic control in patients with type 2 diabetes mellitus (T2DM). DMR appears to improve insulin resistance, which is the root cause of T2DM, but its mechanism of action is largely unknown. Bile acids function as intestinal signaling molecules in glucose and energy metabolism via the activation of farnesoid X receptor and secondary signaling [e.g., via fibroblast growth factor 19 (FGF19)], and are linked to metabolic health. We investigated the effect of DMR and glucagon-like peptide-1 (GLP-1) on postprandial bile acid responses in 16 patients with insulin-dependent T2DM, using mixed meal tests performed at the baseline and 6 mo after the DMR procedure. The combination treatment allowed discontinuation of insulin treatment in 11/16 (69%) of patients while improving glycemic and metabolic health. We found increased postprandial unconjugated bile acid responses (all *P* < 0.05), an overall increased secondary bile acid response (*P* = 0.036) and a higher 12α-hydroxylated:non-12α-hydroxylated ratio (*P* < 0.001). Total bile acid concentrations were unaffected by the intervention. Postprandial FGF19 and 7-α-hydroxy-4-cholesten-3-one (C4) concentrations decreased postintervention (both *P* < 0.01). Our study demonstrates that DMR with GLP-1 modulates the postprandial bile acid response. The alterations in postprandial bile acid responses may be the result of changes in the microbiome, ileal bile acid uptake and improved insulin sensitivity. Controlled studies are needed to elucidate the mechanism linking the combination treatment to metabolic health and bile acids.

**NEW & NOTEWORTHY** Glycemic and metabolic improvements are seen in patients with type 2 diabetes after replacing their insulin therapy with DMR and GLP-1. These changes are accompanied by changes in postprandial bile acid concentrations: increased unconjugated and secondary bile acids.

## Graphical abstract

**Figure:**
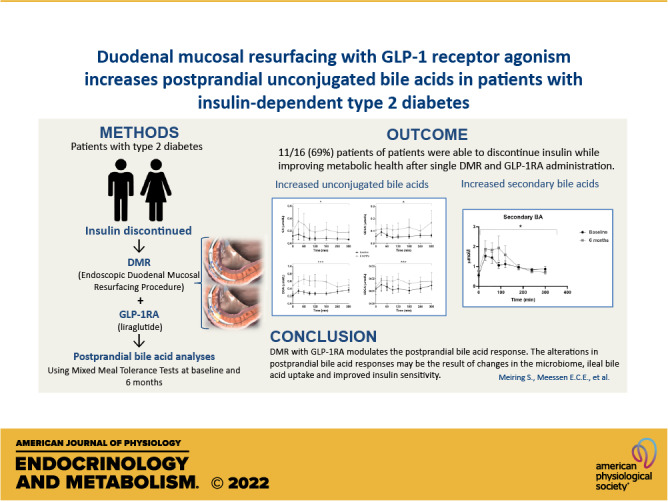

## INTRODUCTION

Globally, the incidence of type 2 diabetes mellitus (T2DM) is increasing and almost 1 out of 10 humans is affected (1). Today, T2DM treatment consists of lifestyle interventions and pharmacological agents but no more than half of all patients with T2DM are able to achieve their treatment goals (2). Bariatric surgery is currently the most effective treatment for T2DM, achieving long-term remission in roughly half of postoperative patients (3–7). Patients undergoing Roux-en-Y gastric bypass surgery (RYGB) demonstrate major improvements in glycemic control and metabolic and cardiovascular health, which occur virtually immediately after surgery and well before any significant weight loss is established (8–10). Duodenal mucosal resurfacing (DMR) is a novel endoscopic procedure that ablates the duodenal mucosa using a hot balloon catheter system. It involves ablation with subsequent regeneration of the duodenal mucosa (11). Data from human and animal studies suggest that DMR has an insulin-sensitizing effect that, to some extent, resembles the metabolic improvements observed after bariatric surgery without its associated weight loss. A recent European multicenter study reported that in patients with T2DM on oral glucose-lowering drugs, a single DMR procedure caused significant improvement in glycemia, insulin resistance, and liver transaminase levels after 6 mo, and throughout 2 yr (12). In this study, most of the postprocedure adverse events (AEs) were mild and self-limiting. The results of a recent multicenter, sham-controlled randomized trial, underlined these findings (13). Mechanism of action behind these glycemic and metabolic improvements after bariatric surgery and endoscopic duodenal interventions such as DMR and EndoBarrier has not been fully elucidated. However, some of the mechanisms are linked to endocrine actions of bile acids (14).

The primary bile acids cholic acid (CA) and chenodeoxycholic acid (CDCA) are synthesized from cholesterol in the liver and conjugated to either glycine (G-) or taurine (T-). The balance between CDCA and CA formation is determined by the activity of CYP8B1, which catalyzes 12-hydroxylation of an intermediate common to both biosynthetic routes. CYP8B1 expression is regulated by insulin through the transcription factor FOXO1 (15). After nutrient intake, bile acids are released into the duodenum and function as fat emulsifiers. Bile acids are either reabsorbed via active transport in the ileum or passively in the colon. In the colon, the primary bile acids are deconjugated and can be converted into the secondary bile acids deoxycholic acid (DCA), lithocholic acid (LCA), and ursodeoxycholic acid (UDCA) by the gut microbiota (16). Hereafter, they reach the liver for resecretion into bile, thus, completing the enterohepatic cycle. Bile acids act as signaling molecules in bile acid, glucose, and energy metabolism via a number of specific (nuclear) receptors including the hepatic and intestinal farnesoid X receptor (FXR) in the small intestine and liver. Direct hepatic and indirect intestinal (via FGF19) FXR activation exert negative feedback on bile acid synthesis, this is reflected in reduced levels of a plasma marker metabolite C4(7α-hydroxy-4-cholesten-3-one). Moreover, the ileal produced FGF19 induces hepatic glycogen synthesis and inhibit gluconeogenesis and lipogenesis (17–19).

Previous studies reported that postprandial bile acid plasma concentrations are altered in patients with obesity and T2DM and are associated with disturbed glucose metabolism (20–24). In addition, both RYGB and placement of a duodenal-jejunal bypass liner (DJBL), are associated with changes in postprandial plasma bile acids and bile acid composition and this has been related to altered enterohepatic cycling of bile acids and gut microbiome composition in the postinterventional state (25–27).

In the current predefined substudy, we investigated the effect of DMR and GLP-1 on fasting and postprandial plasma bile acid responses, FGF19, and C4 in patients with T2DM.

## MATERIALS AND METHODS

### Study Design

This pilot study was a single-center, single-arm, prospective, open-label clinical study that evaluated the effect of a single DMR procedure combined with A glucagon-like peptide-1 receptor agonist [glucagon-like peptide-1 receptor agonist (GLP-1RA; liraglutide)], supported by moderate nutritional and lifestyle counseling in patients with T2DM, previously treated with insulin therapy. During nutritional counseling, patients were instructed by a dietician to adhere to a personalized diet (28). The study protocol was approved by the medical ethics committee of the Amsterdam University Medical Center. The study was conducted in accordance with ICH Good Clinical Practice Guidelines and the Declaration of Helsinki. The study is registered under EudraCT No. 2017-00349-30 at Clinicaltrialsregister.eu. The main clinical outcomes of this study have been reported elsewhere (28).

### Clinical Study Summary

We included 16 patients with T2DM using long-acting insulin, aged 28–75 yr, with a body mass index of 24–40 kg/m^2^, an HbA1c ≤ 8.0% (62 mmol/mol), and an adequate β-cell reserve (defined as fasting C-peptide >0.5 nmol/L) (28).

The endoscopic DMR procedure was performed under deep sedation with propofol by a single endoscopist (J.J.G.H.M.B.) with experience in endoscopic DMR procedures. The DMR procedure involved circumferential ablation of the duodenal mucosa using an over-the-guidewire catheter with a balloon at the tip, as described previously (11, 12). Insulin administration was discontinued immediately after the DMR procedure. Patients were instructed to adhere to a 2-wk postprocedural diet (i.e., gradual transition from liquid to solid food). From that moment on, patients began with self-administration of subcutaneous GLP-1RA, liraglutide (Victoza, Novo Nordisk A/S). Mild nutritional counseling and lifestyle education were provided before DMR and during follow-up (28).

### Mixed Meal Test Protocol

Patients underwent a mixed meal test (MMT) at baseline and at 6 mo after DMR to assess effects on postprandial bile acid/FGF19/C4 response. After an overnight fast, a peripheral cannula was placed in the antecubital vein for the venous blood samples. Fresubin (Fresenius Kabi Nederland BV), consisting of 45% carbohydrates, 16% fat, and 20% protein (200 mL, 400 kcal), was used as standard liquid meal that was completely ingested within 5 min (28).

### Data Collection and Laboratory Analysis

Venous blood samples were drawn just before ingestion at time point (T) 0 min (fasted) and at 30, 60, 90, 120, 180, 240, and 300 min after the ingestion of the liquid meal (28). The blood samples were collected into EDTA vacutainers and directly preserved on ice. Vacutainers were centrifuged for 12 min at 3,000 rpm, and plasma was collected and stored on −80°C until laboratory analyses. Individual bile acid concentrations were measured with an LC-MS assay using a Waters Quattro Premier XE tandem mass spectrometer in the negative electrospray ionization mode, as is published in detail elsewhere (29). FGF19 levels were determined with an in-house developed ELISA. Plasma 7α-hydroxy-4-cholesten-3-one (C4) was assayed by LC-MS (30).

### Calculations and Statistical Analysis

We included bile acid data of all 16 patients in our statistical analysis. Total bile acid concentrations were calculated as the sum of the conjugated (glycine and taurine) and unconjugated forms of CA, CDCA, DCA, LCA, and UDCA. Bile acids below the detection limits of 0.01 µmol/L were assigned a value of 0.005 µmol/L, respectively. If all measurements of an individual bile acids are below the detection limit, this individual bile acid will not be included in the analysis. The primary:secondary bile acid ratio was calculated as the sum of (un)conjugated CA and CDCA concentrations [i.e., plus their conjugates glycocholic acid (gCA), taurocholic acid (tCA), glycochenodeoxycholic acid (gCDCA), and taurochenodeoxycholic acid (tCDCA)] divided by the sum of (un)conjugated DCA, LCA, and UDCA concentrations. The 12α-hydroxylated:non-12α-hydroxylated bile acid ratio was calculated as the sum of (un)conjugated CA and DCA concentrations divided by the sum of (un)conjugated CDCA, LCA, and UDCA concentrations.

Comparisons between the baseline and 6 mo follow-up were assessed with mixed effect models to test for differences between postprandial responses and abovementioned ratios. The intervention was set as fixed effect whereas the time points and patient number were set as random effects. The peak time was defined as the first time point after meal ingestion where the maximal concentration (equals to peak concentration) was reached. The Wilcoxon paired signed-rank test was used to compare fasted concentrations and peak concentrations between the baseline and 6 mo follow-up. We assessed correlations between change in metabolic parameters [ΔHbA1c, Homeostatic Model of Insulin Resistance (HOMA-IR), weight, and liver fat content] and changes (Δ) in individual and grouped postprandial bile acid responses using the Spearman’s ρ test. All data were analyzed using IBM SPSS Statistics 25 (IBM, Armonk, New York). Graphs were created with GraphPad Prism 8 (GraphPad Software Inc., La Jolla, CA). All statistical tests were done two-sided with *P* values < 0.05 considered as statistically significant. Data are presented as median (interquartile range) if not mentioned otherwise. The data in the graphs are presented as means (SE).

## RESULTS

### Patient Characteristics and Clinical Results

As reported previously, patients were on average 61 yr old, had 11 yr of T2DM, and used 31 units of insulin per day before DMR. At 6 mo, 69% of patients (11/16) met the primary clinical end point of the study; that is, off insulin therapy with an HbA1c ≤ 7.5%. Patients also demonstrated significant improvements in metabolic health: body weight, total body fat, and average liver proton density fat fraction (PDFF) decreased significantly. Details on these clinical outcomes have been published elsewhere (28). All LCA measurements were below the detection limit and were excluded from the analyses. Moreover, sample errors occurred in all time points in *patient 2* at the baseline, at time point 300 in *patient 5* at the baseline, and time points 240 and 300 in *patient 11* at the baseline.

### No Changes in Total Bile Acid Concentrations

**Figure 1.**
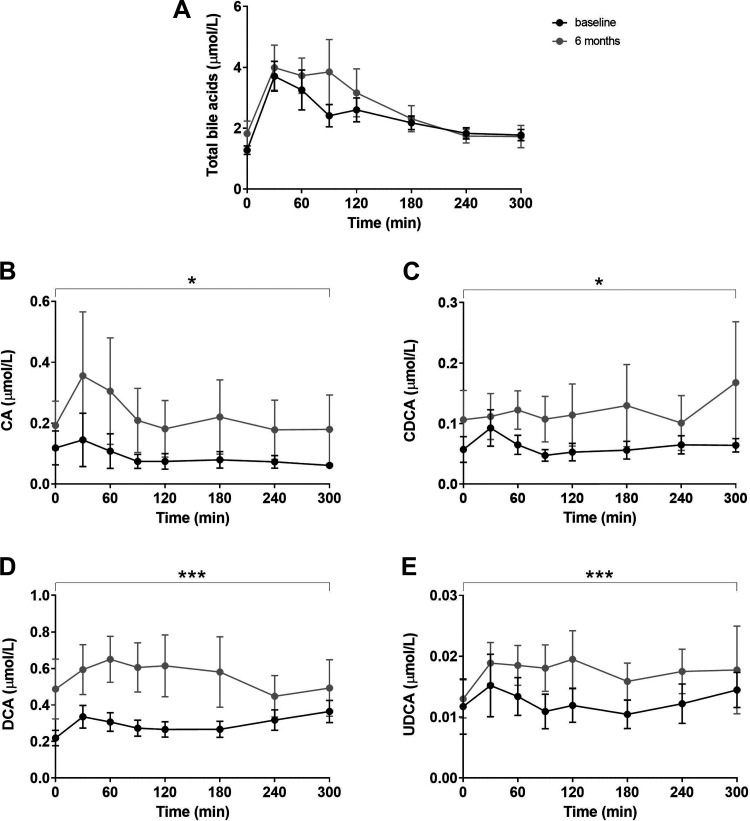
Postprandial excursions of total bile acids (*A*), CA (*B*), CDCA (*C*), DCA (*D*), and UDCA (*E*). Effects of the combination treatment were analyzed with a mixed effect model. *P ≤ 0.05 and ***P ≤ 0.001. Concentrations are presented as means (SE). CA, cholic acid; CDCA, chenodeoxycholic acid; DCA, deoxycholic acid; UDCA, ursodeoxycholic acid.

As expected, ingestion of the test meal resulted in a postprandial elevation of circulating bile acids (Fig. 1*A*). We did not detect differences in fasting concentrations of total bile acids [baseline: 1.19 (0.87) µmol/L vs. at 6 mo: 1.25 (1.01) µmol/L, *P* = 0.69), nor total postprandial bile acids response (Table 1, Fig. 1).

**Table 1.** Bilogram: fasting, peak concentration, and peak times of individual bile acids

	Unconjugated Bile Acids		Glycine Conjugated Bile Acids		Taurine Conjugated Bile Acids	
	Baseline	6 mo	Baseline	6 mo	Baseline	6 mo
	CA		gCA		tCA	
Fasting, µmol/L	0.05 [0.04]	0.04 [0.07]	0.08 [0.10]	0.06 [0.09]	0.05 [0.04]	0.06 [0.03]
Peak, µmol/L	0.07 [0.05]	0.6 [0.06]	0.41 [0.41]	0.24 [0.43]	0.11 [0.19]	0.10 [0.09]
Peak time, min	150 [270]	90 [158]	60 [37.5]	45 [105]	30 [60]	75 [90]
	CDCA		gCDCA		tCDCA	
Fasting, µmol/L	0.02 [0.06]	0.03 [0.04]	0.24 [0.32]	0.23 [0.42]	0.04 [0.03]	0.03 [0.05]
Peak, µmol/L	0.10 [0.15]	0.09 [0.19]	1.17 [0.84]	0.96 [1.47]	0.15 [0.22]	0.14 [0.33]
Peak time, min	240 [248]	60 [45]	30 [30]	60 [60]	30 [60]	75 [90]
	DCA		gDCA		tDCA	
Fasting, µmol/L	0.20 [0.29]	0.27 [0.27]	0.13 [0.25]	0.17 [0.31]	0.03 [0.06]	0.005 [0.061]
Peak, µmol/L	0.44 [0.39]	0.69 [0.63]*	0.83 [0.94]	0.78 [1.72]	0.13 [0.30]	0.12 [0.38]
Peak time, min	240 [270]	60 [68]*	30 [30]	60 [68]	30 [30]	30 [60]
	LCA		gLCA		tLCA	
Fasting, µmol/L			0.02 [0.02]	0.02 [0.02]	0.005 [0.003]	0.005 [0.007]
Peak, µmol/L			0.08 [0.06]	0.11 [0.08]*	0.039 [0.057]	0.054 [0.07]
Peak time, min			60 [68]	60 [38]	75 [120]	60 [150]
	UDCA		gUDCA		tUDCA	
Fasting, µmol/L	0.005 [0.004]	0.01 [0.02]	0.03 [0.05]	0.02 [0.03]	0.005 [0.0]	0.005 [0.0]
Peak, µmol/L	0.016 [0.021]	0.03 [0.03]	0.13 [0.09]	0.09 [0.14]	0.01 [0.01]	0.006 [0.021]
Peak time, min	75 [270]	90 [270]	75 [90]	60 [68]	30 [68]	30 [15]

Concentrations are presented as median [interquartile range]. The unit of fasting and peak concentrations is μmol/L, the unit of peak time is minutes (min). Differences between the baseline and 6 mo visits were assessed using the Wilcoxon signed-rank paired test. *P < 0.05. We did not analyze the postprandial LCA response given the low plasma concentrations of this bile acid. CA, cholic acid; CDCA, chenodeoxycholic acid; DCA, deoxycholic acid; gCA, glycocholic acid; gCDCA, glycochenodeoxycholic acid; gDCA, glycodeoxycholic acid; gLCA, glycodeoxycholic acid; gUDCA, glycoursodeoxycholic acid; LCA, lithocholic acid; tCA, taurocholic acid; tDCA, taurodeoxycholic acid; tCDCA, taurochenodeoxycholic acid; tLCA, taurolithocholic acid; tUDCA, tauroursodeoxycholic acid; UDCA, ursodeoxycholic acid.

### Changes in Fasting Bile Acid Concentrations and Ratios

We did not detect any differences in the individual fasting bile acid responses (Table 1). In addition, neither the absolute fasting concentrations of primary, secondary, conjugated, unconjugated, 12α-hydroxylated, and non-12-α-hydroxylated bile acids differed significantly (data not shown). Fasting conjugated:unconjugated bile acid ratios decreased significantly [baseline: 3.8 (4.36) vs. at 6 mo: 1.82 (1.41) µmol/L, *P* = 0.047]. We did not detect differences in fasting primary:secondary bile acid ratios [baseline: 1.36 (1.07) vs. at 6 mo: 1.18 (1.31) µmol/L, *P* = 0.281] and fasting 12α-hydroxylated:non-12-α-hydroxylated bile acid ratios [baseline: 1.41 (1.30) vs. at 6 mo: 1.54 (0.71), *P* = 0.57].

### Changes in Postprandial Bile Acid Composition

The unconjugated forms of all individual bile acids but LCA, which was not included in the analysis since all measurements were below detection/quantification limits, were found to be significantly increased after 6 mo of combination treatment (Fig. 1, *B*–*E*). The postprandial unconjugated bile acids response of each individual patient can be found in Supplemental Fig. S1 (https://doi.org/10.6084/m9.figshare.14483058.v2).

**Figure 2.**
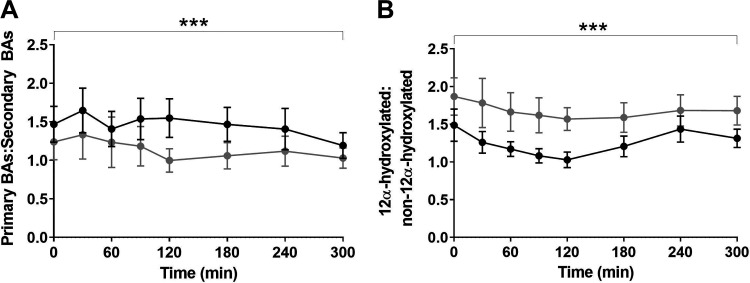
Postprandial excursions of the primary:secondary bile acid ratio (*A*) and hydroxylated:non-12α-hydroxylated bile acid ratio (*B*). The primary:secondary bile acid ratio was calculated by the sum of CA and CDCA (plus their conjugates gCA, tCA, gCDCA, and tCDCA) concentrations divided by the sum of DCA, LCA, and UDCA (plus their conjugates gDCA, tDCA, gLCA, tLCA, gUDCA, and tUDCA) concentrations for all time points. The 12α-hydroxylated:non-12α-hydroxylated bile acid ratio was calculated by the sum of CA and DCA (plus their conjugates gCA, tCA, gDCA, and tDCA) concentrations divided by the sum of CDCA, LCA, and UDCA (plus their conjugates gCDCA, tCDCA, gLCA, tLCA, gUDCA, and tUDCA). Effects of the combination treatment were analyzed with a mixed effect model. ***P ≤ 0.001. Concentrations are presented as means (SE). BAs, bile acids; CA, cholic acid; CDCA, chenodeoxycholic acid; DCA, deoxycholic acid; g-, glycine-conjugated; gCA, glycocholic acid; gCDCA, glycochenodeoxycholic acid; gDCA, glycodeoxycholic acid; gLCA, glycodeoxycholic acid; gUDCA, glycoursodeoxycholic; LCA, lithocholic acid; t-, taurine conjugated; tCA, taurocholic acid; tCDCA, taurochenodeoxycholic acid; tLCA, taurolithocholic acid; tDCA, taurodeoxycholic acid; tUDCA, tauroursodeoxycholic acid; UDCA, ursodeoxycholic acid.

**Figure 3.**
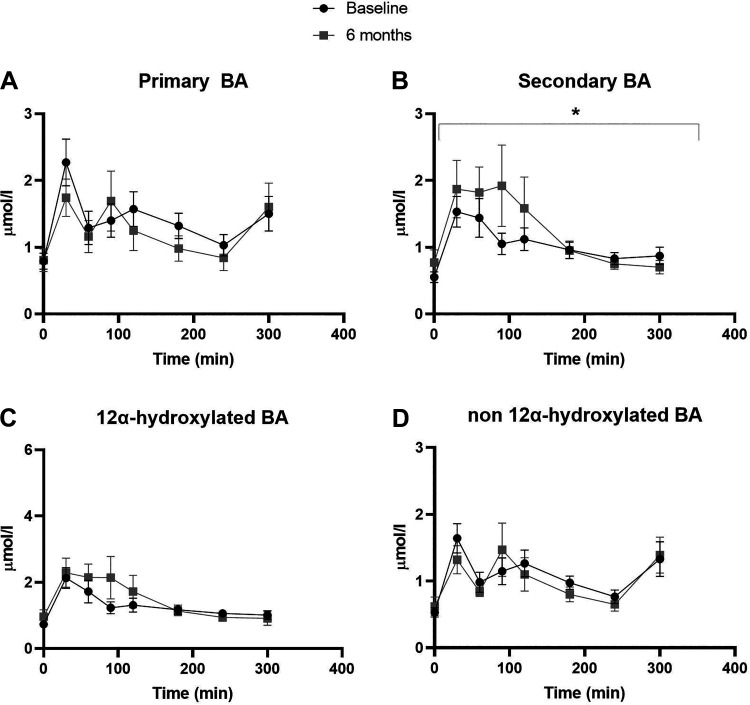
Postprandial excursions of the primary bile acids (*A*), the secondary bile acids (*B*), the 12α-hydroxylated bile acids (*C*) and the non-12α-hydroxylated bile acids (*D*). Effects of the combination treatment were analyzed with a mixed effect model. *P ≤ 0.05. Concentrations are presented as mean (SE). BA, bile acids.

**Figure 4.**
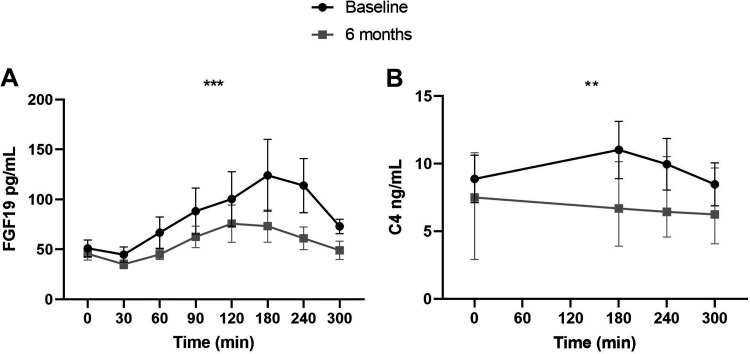
Postprandial excursions of FGF19 (*A*) and C4 (*B*). The unit of fasting and peak concentrations is pg/mL for FGF19 and ng/mL for C4. Effects of the combination treatment were analyzed with a mixed effect model. **P ≤ 0.01 and ***P ≤ 0.001. Concentrations are presented as mean (SE). C4, 7-α-hydroxy-4-cholesten-3-one; FGF19, fibroblast growth factor 19.

The postprandial response of individual glycine- and taurine-conjugated bile acids were changed in ; glycodeoxycholic acid (gLCA) and glycoursodeoxycholic [gUDCA (Supplemental Fig. S2; see https://doi.org/10.6084/m9.figshare.14483052.v4)] and changed in tCA and tCDCA (Supplemental Fig. S3; see https://doi.org/10.6084/m9.figshare.14483055.v3). The ratios of postprandial primary:secondary bile acids were significantly decreased postintervention (*P* < 0.001, Fig. 2*A*). This altered ratio is driven by significantly increased total secondary bile acid responses 6 mo after the DMR treatment (*P* = 0.036, Fig. 3*B*), whereas total primary bile acids concentration did not change (*P* = 0.117, Fig. 3*A*). The ratios of postprandial 12α-hydroxylated:non-12-α-hydroxylated bile acids significantly increased (*P* < 0.001, Fig. 3*B*). Consequently, the postprandial response of total 12α-hydroxylated bile acids increased but did not reach significance (*P* = 0.058, Fig. 4*C*) and total non-12α-hydroxylated bile acids did not change (*P* = 0.241, Fig. 3*D*).

### Minor Changes in Peak Concentrations and Peak Times of Individual Bile Acids

The peak concentrations [baseline: 3.62 (3.65) vs. at 6 mo 3.93 (4.32) µmol/L, *P* = 0.64] and peak times [baseline: 30 (30) vs. at 6 mo: 60 (60) min, *P* = 0.21] of individual bile acids were unchanged. Some individual bile acids showed minor differences in peak concentrations and peak times (Table 1).

### Decreased Postprandial FGF19 and C4 Concentrations

Ingestion of the test meal resulted in elevation of systemic FGF19 levels (Fig. 4*A*), with peak times lagging 2–3 h behind those of total bile acids. The postprandial response of FGF19 was significantly decreased after 6 mo (*P* < 0.001, Fig. 4*A*). Fasting FGF19 concentrations were not affected by the intervention [baseline: 38.1 (47.4) vs. at 6 mo: 38.1 (33.3) pg/mL, *P* = 0.41].

The same pattern was observed for C4: the postprandial C4 response decreased at 6 mo (*P* = 0.002, Fig. 4*B*), whereas fasting C4 concentrations remained unaltered [baseline: 6.3 (9.0) vs. at 6 mo: 7.5 (7.9) ng/L, *P* = 0.20).

### No Correlations between Metabolic Parameters and Postprandial Bile Acid Responses

We did not detect correlations between changes in metabolic parameters (HbA1c, HOMA-IR, weight, and liver fat) and changes in individual bile acids, bile acid ratios, total bile acid concentrations, and FGF19 or C4 concentrations.

## DISCUSSION

DMR is a novel endoscopic treatment involving ablation with subsequent regeneration of the duodenal mucosa (11). Results of the first studies with DMR suggest that a single DMR has an insulin-sensitizing effect, independent of weight loss in patients with T2DM. These studies reported significant improvement in glycemia, insulin resistance, and liver transaminase levels up until 2 yr after the DMR procedure (12). In addition, the procedure was found safe and feasible (11, 12). In our previously reported prospective INSPIRE study, we demonstrated that the combination of DMR and GLP-1RA allowed discontinuing insulin treatment in 69% of the patients while improving glycemic control and metabolic health (28). This clinical study is currently the only study in which postprandial plasma samples of patients were obtained for bile acid analysis to unravel the underlying insulin-sensitizing mechanism of DMR. In the current substudy, we found that postprandial levels of unconjugated bile acids were increased at 6 mo follow-up. We also observed decreased primary:secondary bile acid ratios (i.e., an increase in secondary bile acids) and increased 12α-hydroxylated:non-12-α-hydroxylated bile acids ratios at 6 mo follow-up. Total bile acid concentrations were found to be unaffected, which suggests that there was no big change in bile acid pool size, but rather in composition.

The observed increase in postprandial unconjugated bile acids may result from altered bile acid uptake kinetics in the terminal ileum in patients after DMR. We hypothesize that DMR causes changes in the small intestinal microbiota and consequently the colon microbiota and that these changes influence the expression of the apical sodium bile acid transporter (ASBT). Out et al. (31) have shown that treatment of mice with antibiotics affects expression of ASBT in the small intestine via a GATA4-dependent mechanism suggesting a direct communication between small intestinal bacteria and regulation of ASBT. We have not been able to assess activity of the ASBT, responsible for the bulk of conjugated bile acid uptake (32). However, we hypothesize that the activity of ASBT has decreased, leading to increased spillover of bile acids into the colon followed by increased deconjugation and dehydroxylation by the gut microbiota following DMR (33).

In line with decreased ASBT activity, changes in postprandial bile acid response after DMR induced a reduction in plasma FGF19, indicating decreased intestinal FXR activation. At the same time, the proxy of bile acid synthesis: C4 decreased as well, in turn, indicating increased activation of hepatic FXR. This could be explained by our hypothesis that relatively more unconjugated bile acids reach the colon and are passively reabsorbed into the portal vein, resulting in increased hepatic FXR activation.

Moreover, we found increased 12α-hydroxylated:non-12α hydroxylated bile acid ratios at 6 mo follow-up. This increase could be a result of a tended increase of postprandial 12α-hydroxylated and unchanged non-12α hydroxylated bile acid concentrations. Increased 12α-hydroxylated bile acid concentrations are described in insulin resistance and T2DM in humans (22, 23). The synthesis of the 12α-hydroxylated bile acid CA is regulated by the transcription factor FoxO1 in the liver (15, 34). Insulin inhibits Akt-dependent phosphorylation and nuclear localization of FoxO1. In the setting of insulin resistance, this results in more active FoxO1, followed by higher Cyp8b1 expression which in turn leads to increased CA synthesis (15). Interestingly, our intervention improved insulin sensitivity and exogenous insulin supplementation was discontinued (28). This increase in 12α-hydroxylated bile acid concentrations has also been observed after RYGB, an intervention that also greatly improves insulin sensitivity (35, 36). Moreover, the gut microbiome also plays a role in the dehydroxylation of bile acids (37). This suggests that other, yet unknown, factors also modulate the relation between 12α-hydroxylated bile acids and insulin resistance.

This study has some limitations. First, this is an observational uncontrolled proof-of-concept study with a limited sample size. Causality cannot be proven with this study. Second, due to the design of the study, it is difficult to determine the effect of each individual treatment component. Isolated GLP-1RA administration reportedly increases fasting and postprandial DCA concentrations, whereas other bile acids and total bile acids were unaffected (38). Therefore, GLP-1RA treatment alone cannot explain all of our findings. Third, we used a liquid mixed meal to standardize our mixed meal tests, although it has roughly the same nutritional composition that may have led to different results than using a solid mixed meal (39). Fourth, we cannot exclude the possibility that patients’ intake changed after the DMR. Fifth, human postprandial bile acid responses display significant inter- and intraindividual variability and, therefore, it could be possible that we did not detect all the differences in postprandial bile acid responses (40). Finally, we did not determine 24 h fecal bile acids in this study. This would provide more intel on changes in plasma bile acids. Currently new (controlled) studies are investigating efficacy of hydrothermal ablation (DMR) and other duodenal ablation techniques. So far, mechanistic assessments were only done in open-label studies. It is key to include these assessments (e.g., biopsies for histology, microbiome, and gene and protein expression; mixed meal tests for glucoregulatory hormones; bile acids and metabolomics; and liver biopsies) in randomized setups as well, to elucidate DMR’s mechanism. An overview of the results and hypotheses regarding the mechanism of DMR was recently published (1). The review suggested that DMR might have a place in the T2D treatment ladder in the future. GLP-1RA is already incorporated in diabetes treatment guidelines. When patients have more severe T2D, for example, high insulin resistance, or combined with fatty liver disease, we suggest combining GLP-1 with DMR, because of the apparent positive effects on glycemia and metabolic health of this combination. This article supports evidence that bile acid composition (via FGF19 and FXR) is linked to glycemic control, and that there is a role for bile acids in the mechanism of combined DMR and GLP-1.

### Conclusions

This is the first study to investigate postprandial bile acid responses after a single DMR procedure in combination with GLP-1RA. Our main observations were *1*) increased unconjugated bile acid responses, *2*) increased secondary bile acids responses, and *3*) increased 12α-hydroxylated:non-12-α-hydroxylated bile acid ratios. These observations may result from a change in gut microbiome post DMR but may also result from a change in insulin resistance as a result of DMR. Further trials, studying the underlying mechanism of how DMR alters insulin resistance in patient with T2DM are necessary.

## SUPPLEMENTAL DATA

10.6084/m9.figshare.14483058.v2 Supplemental Fig. S1:https://doi.org/10.6084/m9.figshare.14483058.v2.

10.6084/m9.figshare.14483052.v4 Supplemental Fig. S2:https://doi.org/10.6084/m9.figshare.14483052.v4.

10.6084/m9.figshare.14483055.v3 Supplemental Fig. S3:https://doi.org/10.6084/m9.figshare.14483055.v3.

## GRANTS

This study was supported by Fractyl Laboratories Inc.

## DISCLOSURES

J.J.G.H.M.B. received research support from Fractyl Laboratories Inc. for IRB-based studies and received a consultancy fee for a single advisory board meeting of Fractyl in September 2019. None of the other authors has any conflicts of interest, financial or otherwise, to disclose.

## AUTHOR CONTRIBUTIONS

A.C.G.v.B, F.H., M.N., A.K.G., M.R.S., and J.J.G.H.M.B. conceived and designed research; S.M. performed experiments; S.M., E.C.E.M., A.C.G.v.B., and F.G.S., and J.J.G.H.M.B. analyzed data; S.M., E.C.E.M., F.G.S., and J.J.G.H.M.B. interpreted results of experiments; S.M. and E.C.E.M. prepared figures; S.M. and E.C.E.M. drafted manuscript; S.M., E.C.E.M., A.C.G.v.B., F.H., M.N., S.W.O.D., F.G.S., F.M.V., and A.K.G., M.R.S., and J.J.G.H.M.B. edited and revised manuscript; S.M., E.C.E.M., A.C.G.v.B., F.H., M.N., S.W.O.D., F.G.S., F.M.V., A.K.G., M.R.S., and J.J.G.H.M.B. approved final version of manuscript.
